# Psorinum*Kansas State Homœopathic Society, 1896.

**Published:** 1896-07

**Authors:** J. H. Allen

**Affiliations:** Logansport, Ind.


### PSORINUM.*

J. H. Allen, M. D., Logansport, Ind.

Psorinum comes down to us through the files of the ages, and is known as Psora. Hahnemann says it is recorded in the third book of the Bible, hence it seems to have been one of the first subversive forces manifesting itself upon that degenerate being, man. Some have gone so far as to say it was the primary physical manifestation of original sin ; but be that as it may, it has become recognized as a positive fact, a living reality, by all honest and unbiased minds in all branches of the homœopathic school, and there is only an individual here and there opposing the psoric theory with any degree of opposition. The proclamation of Hahnemann has gone out into all the world that

---

* Kansas State Homœopathic Society, 1896.

such a subversive force exists in the organism, and that it is the primary disturbing principle of biological law, the basic principle of most chronic diseases recorded in our nomenclature, and known as specified pathological states and conditions in our literature; when we as honest men of science begin to investigate *The Organon* and the teachings of Hahnemann the whole psoric theory unfolds itself to us so that no man can be mistaken, or have any hesitancy in accepting the truths as recorded by the master. I will not take up your time by theorizing as to how these artificial subversive forces, known as remedies, act on the natural or diseased life forces, except to say it is only through the law of similars, in which we all believe no matter how limited our knowledge is of the science. Nor have I time to give you my theories as to how a high potency of this natural or diseased subversive force acts subversively upon itself, save to say that the very means of grace exists in the very means of death. In the crude state psora is the arch enemy of true biological law, the penalty of which is death. But the potency when applied through the similar law is the gateway of life, for similia is grace just as much so as if the Christ reached down the finger of His mighty power and touched you and made you whole, for law is suspended through the higher power of grace.

The remedy Psorinum has been proven extensively by Hahnemann, Hering, and others, besides many clinical symptoms have been added, thereby making it of still greater value to the prescriber. It has a very marked and peculiar sphere in the organism, finding its expression in the vital force itself. What would not be considered a symptom under any other remedy may be taken as very characteristic of Psorinum.

Psorinum expresses itself in the lack of vital energy, a lack of response to remedies, foods, or anything seemingly well indicated in the case; lack of reactive power, especially in acute diseases, as in prolonged febrile states. When the patient reaches the reactionary period in disease he does not react promptly, but remains lethargic or in a state of great physical prostration. Often a dose of Psorinum will here arouse the failing vital force which is so hampered by the presence of psora that it cannot respond. A single dose often loosens the life forces from its bonds and it at once sets to work to restore the organism to health. This wonderful action is only known to those who have the pleasure of using this drug. Often with its help you pull your patient out of very difficult corners, and that without the aid of stimulants, or the thousand and one abominations usually employed in such cases. The heart, the circulation, together with all the organs, right themselves in their order as soon as the life forces are freed from the thraldom of psora. The other symptoms peculiar to this remedy might be summed up in the eruptions of the skin, the prostration of body and mind, the great distress in breathing, the cardiac weakness, and more particularly the weakness that cannot be localized in any part of the body in constitutional troubles.

In the mind we have utter hopelessness, melancholy, sadness, even to a fear of death; fear that he will lose his fortune, or that he will die in the poor-house, or become a beggar; if he is of a religious temperament he will despair of his salvation. He has no surplus energy to throw away; he is always ready to lie down when an opportunity offers itself; he is relieved by so doing, also by warmth, rest, quiet. The life forces have all they can do to keep the feeble spark of life burning, and have therefore no power to store up energy on the one hand, nor heat on the other, hence he must have it artificially supplied either by a surplus amount of clothing, which seems greatly out of reason, or by direct artificial heat. These patients are nervous, restless, and easily startled; they are sleepless frequently from intense itching of the body, with burning after scratching. He takes cold at the slightest exposure, and is excessively sensitive to all atmospheric changes or fall of barometer; hence he is better in summer and worse in winter; also much better when perspiring (the reverse of Sulphur; Medorrhinum is also worse in winter). Psorinum is also worse at night.

The psoric baby cries, frets, whines all night pitifully (Jalap); it has no assimilation, hence nothing agrees with it, not even water; the body smells foul all the time, and it is not removed by washing; it has pimply and scaly eruptions on any part of the body, and all discharges and excretions smell foul, carrion-like, offensive—stools, expectoration, breath, menses, leucorrhoea, everything offensive.

Hunger is a marked symptom in many cases, even ravenous hunger; eating often relieves the headache. Headaches are also made better by washing the face, by profuse sweat, and by nose-bleed. Hunger at night, he wakes up hungry; his face is usually pale, sallow, thin, sickly looking.

The hair is dry, easily tangled, glues together, lustreless (*plica polonica*); he has an aversion to having the head uncovered, and wears a heavy cap when he should wear a straw hat; sensitive to the least draft, even in bed. Frequently we find raw, moist sores around the margin of the hair exuding an offensive secretion, often pus; scurfy, scaly scalps in children; copious dandruff, brown, bran-like scales, and when washed out soon form again; itching when warm, burning after scratching; rawness and soreness behind the ears (like Hepar, Calc-c., Sil., Petroleum); itching boils in the scalp exuding offensive pus and bleeding easily (like Nit-ac., Hepar, Merc.).

Papular eruptions in the flexures of the joints, between the fingers, burning worse at night and from warmth of bed; sweats easily; grows weak and then, no matter what the trouble is, has much anxiety about his health; scratches till the blood comes, when he falls asleep; the skin trouble is worse undressing (like Hepar, and others); children bury their faces in the pillows in bronchial troubles, which are accompanied with thirst and rattling cough. In the eyes and eyelids we have many prominent symptoms of this remedy worthy our attention. The lids are inflamed, red like raw beef, scaly, scurfy crusts on edges; eyelashes gone entirely or else stuck together (Merc., Graph., Cinnabaris, Sulph.); worse in the open air and better by warmth of room, with itching and burning after using them; desires to keep them closed or else to rub them.

In the ears we have all sorts of noises; singing, cracking, humming, buzzing, often with an offensive discharge of watery pus or reddish cerumen; bores and picks in the ears; scaly, bran-like eruption in the meatus.

The pains of this remedy, whether in the stomach or any organ of the body, are of a burning nature usually, especially after suppressed eruptions or suppressed conditions from which chronic dyspepsia is apt to develop in business or railroad men who eat hurriedly, and who resume their work soon after eating; they usually are hearty eaters. The eructations smell like rotten eggs, or there is vomiting of sour mucus before eating, with weakness in the stomach and backache in the morning, or with oppression after eating. Stools in diarrhœa are usually preceded by colic, and are usually thin, watery, greenish, or brownish, bilious and united with mucus; hot, fetid, very offensive, driving you out of the room, like rotten eggs or hydrogen sulphide; usually painless and worse at night or change of weather. Skin often unhealthy, dirty, greasy, covered with a poorly-developed eruption. Cholera infantum in children who cry and fret all night, no peace with them till morning, when they fall into an exhausted sleep.

Talking fatigues, voice weak, chronic hoarseness in psoric patients or in tubercular patients; suffocating, crawling sensation in the larynx producing a dry paroxysmal cough (Tuberculinum) as if the throat were narrow. Hay fever; often this is the best remedy with which to begin treatment. The chest in these patients expands with difficulty; they have anxious dyspnœa in lung and bronchial troubles, with palpitation and pain in the region of the heart. Asthma in psoric patients, with dry, hard cough, caused usually by tickling in the larynx; worse sitting and in the morning. Such cases have been known to last for thirty years before the lungs broke down. Suppressed eruptions are the cause of the most aggravated forms of psora. The expectoration is frequently profuse and greenish, tastes salty; all chest symptoms are better by lying down.

The chill generally begins in the arms or thighs, usually in the evening, with much thirst, but drinking causes cough; pain in the chest follows the heat, the fever is then followed by a profuse, foul-smelling perspiration, copious, trickling down the face, clammy and sticky. There is usually a tired feeling before the chill, with a desire to lie down and to stretch out the arms, and a desire to cover up, though they are perspiring profusely and the temperature is high.

The cough is always better by lying down ; also chest symptoms. Weakness in all the joints ; often trembling of the hands and feet. Rheumatism of the extremities with suppressed eruptions, or following suppressed skin troubles ; or gouty pains with dry cough accompanied by ill-humor and despondency, with heat in hands or feet ; similar to Sulphur. All troubles relieved by reproducing the suppressed eruption, and cured by Psorinum.

